# Differential Modulation of *I_K_* and *I_Ca,L_* Channels in High-Fat Diet-Induced Obese Guinea Pig Atria

**DOI:** 10.3389/fphys.2019.01212

**Published:** 2019-09-25

**Authors:** Laura Martinez-Mateu, Javier Saiz, Ademuyiwa S. Aromolaran

**Affiliations:** ^1^Centro de Investigación e Innovación en Bioingeniería, Universitat Politècnica de València, Valencia, Spain; ^2^Cardiac Electrophysiology and Metabolism Research Group, VA New York Harbor Healthcare System, Brooklyn, NY, United States; ^3^Department of Cell Biology, State University of New York Downstate Medical Center, Brooklyn, NY, United States; ^4^Department of Physiology & Cellular Biophysics, Columbia University, New York, NY, United States

**Keywords:** high-fat diet, hERG, KCNQ1, cardiomyocytes, guinea pig, atria

## Abstract

Obesity mechanisms that make atrial tissue vulnerable to arrhythmia are poorly understood. Voltage-dependent potassium (*I_K_*, *I_Kur_*, and *I_K1_*) and L-type calcium currents (*I_Ca,L_*) are electrically relevant and represent key substrates for modulation in obesity. We investigated whether electrical remodeling produced by high-fat diet (HFD) alone or in concert with acute atrial stimulation were different. Electrophysiology was used to assess atrial electrical function after short-term HFD-feeding in guinea pigs. HFD atria displayed spontaneous beats, increased *I_K_* (*I_Kr_ + I_Ks_*) and decreased *I_Ca,L_* densities. Only with pacing did a reduction in *I_Kur_* and increased *I_K1_* phenotype emerge, leading to a further shortening of action potential duration. Computer modeling studies further indicate that the measured changes in potassium and calcium current densities contribute prominently to shortened atrial action potential duration in human heart. Our data are the first to show that multiple mechanisms (shortened action potential duration, early afterdepolarizations and increased incidence of spontaneous beats) may underlie initiation of supraventricular arrhythmias in obese guinea pig hearts. These results offer different mechanistic insights with implications for obese patients harboring supraventricular arrhythmias.

## Introduction

High-fat diet (HFD)-induced obesity is associated with insulin resistance, Type 2 diabetes mellitus (T2DM), and dyslipidemias (Schulze et al., 2016). A recent estimation by the National Institutes of Health (NIH) shows that obesity and its co-morbidities, affects 17% of children and young adults in the United States, while over one-third of adults are overweight and/or obese (Jensen et al., 2014). Moreover, obesity is an independent and key contributor to the expanding prevalence of atrial fibrillation (AF) (Michael et al., 2009; Lau et al., 2012; Abed and Wittert, 2013), a serious condition affecting approximately 2.7 million people in the United States (www.heart.org/en/health-topics/atrial-fibrillation).

In population-based cohort studies, obese individuals showed a 49% increase in vulnerability to AF when compared to non-obese individuals (Wanahita et al., 2008). A third wave of the Nord-Trøndelag Health prospective cohort study (HUNT3), demonstrated that being overweight or obese increased the AF risk by 18 and 59% (Garnvik et al., 2018); while epidemiological studies revealed a 4–5% increased risk of AF for each one unit increase in body mass index (BMI) (De Sensi et al., 2018). Despite the growing knowledge that obesity and AF are serious conditions with significant implications for public health, the molecular mechanisms that underlie atrial remodeling in obesity are poorly understood.

Atrial electrical remodeling due to pathological changes in functional expression of major atrial ion channels is an important signature of AF initiation (Aromolaran and Boutjdir, 2017; Dan and Dobrev, 2018; Heijman et al., 2018; Rahm et al., 2018). The human atrial action potential (AP) is defined by: phase 0 controlled by a large inward sodium current (*I_Na_*) (Ni et al., 2017; Pezhouman et al., 2018), followed by calcium entry through L-type calcium channels (*I_Ca,L_*) due to Ca_v_1.2/Ca_v_1.3 channels (Barana et al., 2014); a plateau phase maintained by a balance between inward and outward currents. Repolarization is controlled by fast transient outward potassium currents (*I_to_*), ultra-rapid (*I_Kur_*), rapid (*I_Kr_*), and slow (*I_Ks_*) delayed rectifier currents (Nerbonne and Kass, 2005). The resting membrane potential is determined by the inwardly rectifying potassium currents (*I_K1_*) (Ji et al., 2017; Whittaker et al., 2017).

Therefore, altered ion channel function that either increases potassium currents (Aromolaran et al., 2016) or decreases calcium currents (Van Wagoner et al., 1997, 1999; Christ et al., 2004; Mancarella et al., 2008) would be expected to promote an accelerated repolarization process, leading to shortened action potential duration (APD) (Boutjdir et al., 1986; Aromolaran et al., 2016), atrial refractoriness (Boutjdir et al., 1986), leading to ectopic firing (Nattel, 2002; Iwasaki et al., 2011; Czick et al., 2016) and re-entry mechanisms (such as single or multiple wave patterns) (Wakili et al., 2011; Nattel and Dobrev, 2017). To advance the current knowledge of AF mechanisms and development of mechanism-based therapeutic interventions, future studies will require robust approaches including coupling of experimental data with computational modeling (Grandi et al., 2011, 2019; Bai et al., 2018; Martinez-Mateu et al., 2018).

Obesity mechanisms may have a direct impact on the electrical activity of the heart. How the relative functional expression of potassium and calcium channels is altered in HFD-induced obesity remains unknown. It is known that chronically elevated levels of free-fatty acids (FFAs) is associated with obesity-related pathological changes (including insulin-resistance, hyperglycemia, T2DM, and inflammation) (Sonnenberg et al., 2004; Boden et al., 2005; Matafome and Seica, 2017; Sharma et al., 2017). Therefore, hyperlipidemia may represent a common link among obesity and its comorbidities and increased vulnerability to arrhythmias (Chiu et al., 2001; Rennison and Van Wagoner, 2009; Djousse et al., 2013; Fretts et al., 2014).

Furthermore, unlike the monounsaturated oleic acid (OA), saturated free-fatty acids including palmitic acid (PA) (O’Connell et al., 2015; Aromolaran et al., 2016), have been shown to promote adverse ion channel function and are prime candidates for mediating obesity-related adverse electrical remodeling leading to arrhythmias. Therefore, understanding lipotoxicity as a key obesity mechanism is an essential part of the wider effort to understand atrial electrical remodeling in obese patients with arrhythmias.

Unlike rodent models (Matsuura and Ehara, 1997; Nakaya et al., 2000; Killeen et al., 2008a,b), guinea pigs display robust expressions of *I_Kr_* and *I_Ks_* (Hume and Uehara, 1985; Aoki et al., 2012; Aromolaran et al., 2014, 2016). To date there have been increasing number of studies utilizing guinea pig atrial myocytes (Baro and Escande, 1989a,b; Inoue et al., 1993a,b; Bunemann et al., 1996; Bosch et al., 2003; Ochi et al., 2006; Zankov et al., 2006; Suzuki et al., 2014), to understand adverse ion channel mechanisms in disease states. Similar to humans, guinea pigs have high low-density lipoprotein/high-density lipoprotein (LDL/HDL) ratios, express key enzymes involved in lipoprotein metabolism (Fernandez et al., 1995; Sharman et al., 2008), and have higher concentrations of hepatic free cholesterol versus esterified cholesterol ratio (Angelin et al., 1992; Roy et al., 2000). These properties in addition to their use as models of arrhythmias (Laurita and Rosenbaum, 1997; O’Hara and Rudy, 2012; Liu et al., 2014; Mickelson and Chandra, 2017; Dey et al., 2018), further underscores the suitability of using obese guinea pigs in our studies.

In the present study we investigated the effect of HFD on the electrophysiological properties of major atrial ion channels, namely: the delayed rectifier potassium current *I_K_* (or *I_Kr_* + *I_Ks_*), *I_Kur_*, *I_K1_*, and *I_Ca,L_* currents in guinea pig atrial myocytes and determined its role in atrial arrhythmogenesis. Our data show increased *I_K_* and decreased L-type calcium channel densities in atrial myocytes from HFD-fed guinea pigs, whereas *I_Kur_* and *I_K1_* densities were unchanged. The data suggest that these changes are the cause of atrial arrhythmogenesis and therefore may underlie key electrical events (accelerated repolarization, shortened action potential duration, atrial refractoriness, conduction abnormalities, ectopic firing and single/multiple wave re-entrant mechanisms) that lead to AF onset and/or its maintenance in obesity.

## Methods

### High-Fat Diet Feeding in Guinea Pig Model

Guinea pigs (male/female; 200–250 g) were purchased from Charles River Laboratories (Wilmington, MA). Guinea pigs were randomly separated into two groups namely: control and overweight/obese. Controls were fed, *ad libitum*, a low-fat diet (LFD, Research Diets Inc., New Brunswick, NJ USA) containing (in kcal%): 10 fat, 70 carbohydrates, 20 protein, and 2,300 corn starch. HFD groups were fed a palatable high sucrose diet (in which most of the corn starch, was replaced with 1,014 kcal% sucrose), containing 45% of its kcal from fat, 35% from carbohydrates, and 20% from protein. The HFD was calorically rich (4.21 kcal/g versus 3.49 kcal/g for LFD) due to its higher fat content mostly from lard. HFD contained saturated and unsaturated free fatty acids (FFA), which provided 31.4 and 68.6% of the fat-derived calories (Caillier et al., 2012; Patoine et al., 2013). A detailed description and composition of the diets has previously been provided by Drolet’s group (Caillier et al., 2012). Guinea pigs were fed LFD or HFD for a duration of 50 days (~7 weeks), while monitoring temporal changes in weight 2–3 times weekly.

### Isolation of Guinea Pig Atrial Myocytes

Guinea pig atrial myocytes were isolated as previously described (Aromolaran et al., 2016; Puckerin et al., 2016). Briefly, adult male and female Hartley guinea pigs were deeply anesthetized with isoflurane in accordance with the guidelines of the Columbia University Animal Care and Use Committee. Excised hearts were Langendorff perfused with Tyrode solution containing (in mM): 118 NaCl, 4.8 KCl, 1 CaCl_2_, 10 glucose, 1.25 MgSO_4_, and 1.25 K_2_HPO_4_ (pH = 7.4) for 5 min. The heart was then perfused with Ca^2+^-free Tyrode solution for 10 min before switching to Ca^2+^-free Tyrode solution containing Collagenase B (final concentration, 0.6 mg/ml; Boehringer Mannheim, Indianapolis, IN) for an additional 10 min. The heart was subsequently perfused with high K solution containing (in mM): 70 KOH, 50 L-glutamic acid, 40 KCl, 20 Taurine, 20 KH_2_PO_4_, 3 MgCl_2_, 10 glucose, 10 HEPES, and 1 EGTA (pH 7.4, with KOH), for 5–10 min. The digested tissue was placed in fresh high K solution, minced into smaller pieces, and then re-suspended several times to dissociate the cells. The cell suspension was filtered through a mesh and allowed to settle for 15–20 min. The pellet was resuspended in M199 media supplemented with 10% fetal bovine serum (FBS) and then plated on laminin-coated coverslips. Media were replaced with M199 containing 1% FBS 1–3 h after initial plating before experiments. All experiments were performed at room temperature (23–25°C).

### Generation of hERG Plasmid Constructs

hERG 1a and hERG 1b constructs were a gift from Dr. Gail Robertson (University of Wisconsin). Yellow fluorescent protein (YFP) and cyan fluorescent protein/probe (CFP) were fused in frame to the C-terminus of hERG 1a or 1b using overlap extension PCR. A high-affinity 13 amino acid (WRRYYESSLEPYPD) surface epitope bungarotoxin-binding sequence (BBS) (Aromolaran et al., 2014) was introduced between amino acid residues 177–178 and 517–518, located within the extracellular loop of, respectively, hERG 1a S3-S4 domains using a QuikChange Lightning Site-Directed Mutagenesis Kit (Stratagene, La Jolla, CA) according to the manufacturer’s instructions. All cDNA plasmids were sequenced to verify that no error was introduced during preparation.

### HEK293 Cell Culture and Transfection

Low-passage-number HEK293 cells (ATCC, Manassas, VA, USA) were maintained in DMEM supplemented with 10% FBS and 100 μg ml^−1^ penicillin-streptomycin at 37°C. In all experiments, cells were transiently transfected with 1 μg T-antigen and a total of 6 μg of the respective DNA plasmids, using the calcium phosphate precipitation method. For flow cytometry assays, hERG channel subunits were transfected in 1:1 (or 3:3 μg, BBS-hERG 1a-YFP + 1b-CFP or hERG 1a-YFP + 1b-CFP) ratio. Cells were cultured in supplemented DMEM at 37°C for 24 h before transfection.

### Preparation of Bovine Serum Albumin-Conjugated Free Fatty Acid Solutions

Palmitic acid (PA) and oleic acid (OA) were prepared as previously described (Aromolaran et al., 2016). FA-free (20%) bovine serum albumin (BSA, Roche) was dissolved in DPBS. PA and OA (Sigma-Aldrich, St. Louis, MO, USA) were dissolved in ethanol to produce a stock solution of 0.2 M fatty acid. 20% BSA and 0.2 M FA were then mixed in 20:1 volume ratio. Desired concentrations of PA or OA were prepared from ~10 mM fatty acid stock solutions, in M199 culture media or Tyrode’s solution. The control solution was prepared with BSA, ethanol, and DPBS.

### Electrophysiology

Patch clamp experiments in adult guinea pig atrial myocytes were performed at room temperature (20–25°C), using an EPC-10 patch clamp amplifier (HEKA Electronics) controlled by PatchMaster software (HEKA) as described (Puckerin et al., 2016). Briefly, coverslips with myocytes were placed on the stage of an inverted microscope (Eclipse T*i*-U, Nikon). The internal solution contained (in mM): 130 KCl, 1 MgCl_2_, 0.4 GTP, 5 EGTA, 5 K_2_ATP, and 10 HEPES (pH 7.2). External solution contained (in mM): 137 NaCl, 4 KCl, 1.8 CaCl_2_, 1 MgCl_2_, 10 glucose, and 10 HEPES (pH 7.4). Pipette resistance was typically 1.5–2 MΩ and series resistance was compensated 80–90% before each recording. Membrane potentials were corrected for liquid junctional potential. AP’s were recorded from single atrial myocytes in current clamp mode by passing depolarizing currents for 20 ms at subthreshold (1.5×) intensity. Population *I*–*V* curves for *I_K_* were generated by step depolarizations (−40 to +100 mV), from a holding potential of −50 mV in 10 mV increments for 3 s, followed by a repolarizing step to −50 mV for 1 s, with each pulse repeated every 10 s. The external solution contained nifedipine (5 μM), to block Ca currents. *I_Kur_* was studied with a protocol consisting of a 300 ms depolarizing pulses from a −40 to +100 mV from a holding potential of −50 mV in 10 mV increments, followed a repolarizing step to −50 mV. *I_K1_* was activated from −40 mV to test potentials ranging from −120 to +10 mV in 10 mV steps for 1 s. *I_Kur_* and *I_K1_* currents were measured in an external solution contained chromanol 293B (100 μM), E4031 (5 μM), and nifedipine (5 μM) to block *I_Ks_*, *I_Kr_*, and *I_Ca,L_*, respectively. The internal solution for *I_Ca,L_* recordings contained (in mM): 18 CsCl, 108 Cs-aspartate, 1.2 MgCl_2_, 11 HEPES, 1 EGTA, 10 glucose, and 2 MgATP (pH = 7.2, adjusted with Tris). External solution contained (in mM): 6 CsCl, 140 NaCl, 10 glucose, 1 MgCl_2_, 2 CaCl_2_, and 5 HEPES (pH = 7.4 adjusted with NaOH). The *I_Ca,L_* was activated by a series of 250-ms depolarization pulses from −90 mV holding potential to test potentials ranging from −40 to +60 mV (10 mV step) at 10-s intervals. Currents were sampled at 20 kHz and filtered at 5 or 10 kHz.

### Epicardial Electrograms From Isolated Hearts

LFD- or HFD hearts were Langendorff-perfused with Krebs-Henseleit buffer solution containing the following (in mM): 118 NaCl, 4.7 KCl, 1.2 KH_2_PO_4_, 1.2 MgSO_4_, 1.8 CaCl_2_, 25 NaHCO_3_, and 11.1 glucose, (pH 7.4), bubbled with 95% O_2_–5% CO_2_ at 35 ± 1°C. An eight-channel amplifier (Powerlab System, AD instruments, Oxfordshire, United Kingdom) was used to record epicardial atrial electrical activity using two chlorinated electrodes that were placed on the heart (Mancarella et al., 2008). A third module was a stimulator that was controlled by a computer which was designed to deliver programmed fast burst stimulation protocol (rectangular pulses 10 ms in duration, 1 V in amplitude with various pulse-pulse duration 23–190 ms), followed by three extra-stimuli S_1_-S_2_-S_3_ delivered at 300 beats/min, with S1 (250 ms for 50 beats); S2 (50 ms for 100 beats); S3 (20 ms for two beats). The stimuli were delivered to the right atrium to induce atrial arrhythmogenesis or arrhythmia.

### Quantification of Cell Surface Bungarotoxin-Binding Sequence-hERG-Yellow Fluorescent Protein Channels With Quantum Dots

Relative surface expression of BBS-tagged hERG subunits was quantitatively determined using quantum dot labeling or used in flow cytometry (using a BD LSRII Cell Analyzer) to determine the BBS-tagged hERG 1a-YFP or hERG 1b-YFP surface density as described previously (Aromolaran et al., 2014).

### Confocal Imaging

HEK293 cells were imaged using a Zeiss LSM 510 META scanning confocal microscope. CFP and YFP fluorescence signals were captured at, respectively, 458 and 514 nm argon laser lines for excitation and the red fluorescence signals of quantum dot were imaged using the 633 nm helium-neon laser line for excitation. Eight-bit images were obtained using identical laser powers, photomultiplier gain, and pinhole size.

### Computer Simulations

**Figure 1:**
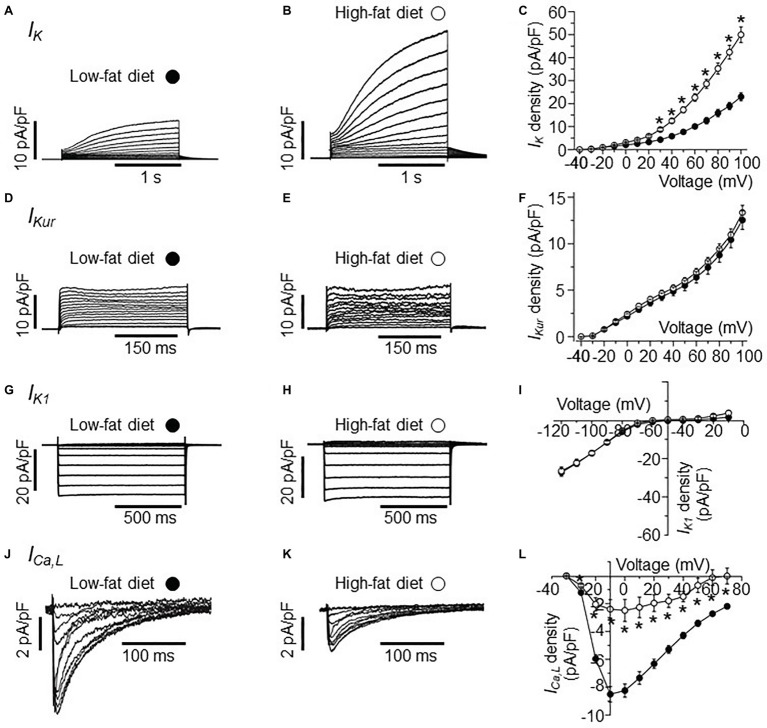
Electrophysiological properties of *I_K_*, *I_Kur_*, *I_K1_*, and *I_Ca,L_* currents in HFD atrial myocytes. **(A)** Representative *I_K_* traces from right atrial myocytes isolated from LFD-fed guinea pigs. **(B)** Exemplar *I_K_* currents measured in HFD atrial myocytes. **(C)** Population *I-V* curves for peak *I_K_* currents measured in LFD (●, *n* = 10) or HFD (○, *n* = 9) atrial myocytes. Exemplar currents and *I*-*V* relationships for indicated *I_Kur_* **(D–F)**, *I_K1_* **(G–I)**, and *I_Ca,L_* **(J–L)**, same format as **(A–C)**. Data points are means ± S.E.M, *n* = 5–20. ^*^p < 0.05. Data were generated from three different guinea pig cardiomyocyte preparations.

We used computer simulations in order to study in human atrial myocytes the effects of the experimentally observed effects of HFD-induced obesity in guinea pig. For this purpose, we simulated the electrical activity of a human atrial myocyte corresponding to a non-obese individual (or LFD) using ionic models described by Courtemanche et al., (1998) and Koivumäki et al. (2011). Then, we modified several ionic currents based on the changes measured in our experiments within the voltage range of the AP, i.e., between −80 and +40 mV (see Table 1). Within this voltage range, for HFD myocytes, I_Kr_ and I_Ks_ were increased by 100% and *I_Ca,L_* was decreased by 70%, while for HFD myocytes after pacing only *I_CaL_*, *I_K1_* and *I_Kur_* were modified with respect to LFD myocytes (*I_CaL_* and *I_Kur_* decreased by 40% and *I_K1_* increased by 112%). Other variations experimentally observed were not considered since they were significantly out of the voltage range in which the AP is contained, as shown in Figures 1, 3. As the computational models differentiate between *I_Kr_* and *I_Ks_*, same variation was applied to both currents to account for the *I_K_* measurements.

**Table 1:** Currents modified in the HFD myocyte, with and without pacing, with respect to the low-fat diet myocyte used for human atrial myocyte simulation models.

Atrial currents	LFD	HFD	HFD + pacing
*I_Ca,L_*	1	0.30^c^	0.60^c^
*I_Kr_*	1	2.00^a^	1
*I_Ks_*	1	2.00^a^	1
*I_K1_*	1	1	2.12^d^
*I_Kur_*	1	1	0.60^b^

*Values are relative to the Courtemanche or Koivumäki models (Courtemanche et al., 1998; Koivumäki et al., 2011). Relative values based on our experimental measurements of: ^a^I_K_ at V = 40 mV; ^b^I_Kur_ at V = 40 mV; ^c^I_Ca,L_ at V = −10 mV; ^d^I_K1_ at V = −80 mV*.

### Data and Statistical Analyses

Electrophysiological data were analyzed off-line using built in functions in Fitmaster (HEKA), and Origin software. Currents are either expressed as current densities (pA/pF). Time constants for current activation, were obtained by fitting the data to a single exponential function. Time constants of rate of *I_K_* tail current deactivation, measured at −50 mV was fitted to a single exponential function of the form *y* = *y*_0_ + *A*_1_e^(−*x*/*t*)^, where *t* is the time. Data are reported as means ± S.E.M. For all electrophysiology and biochemistry assays, statistical differences were obtained from one-way ANOVA with Bonferroni *post hoc* analysis or two-tailed unpaired *t* test for comparisons between groups and considered significant at *p <* 0.05.

## Results

### Effects of High-Fat Diet on *I_K_* and *I_Ca,L_* in Guinea Pig Right Atrial Myocytes

In the initial set of experiments, we investigated the effects of HFD on *I_K_* current measured in atrial myocytes isolated from HFD-fed guinea pigs compared to LFD-fed controls using whole-cell patch clamp (Figure 1). Compared to *I_K_* measured in freshly isolated atrial myocytes from LFD-fed controls (Figure 1A), HFD feeding significantly increased the *I_K_* density (Figure 1B) at all potentials positive to +20 mV (Figure 1C). The data is in line with our previous finding that atrial *I_K_* density is significantly increased by HFD feeding in guinea pigs (Aromolaran et al., 2016). At +100 mV, *I_K_* peak density was increased by 115% (from 23.52 ± 1.32 pA/pF, *n* = 10, to 50.7 ± 3.45 pA/pF, *n* = 9, ^*^p < 0.05, Figure 1C, see Table 2).

**Table 2:** Comparison of averaged ion current densities measured in atrial myocytes isolated from LFD- and HFD-fed guinea pigs after 50 days.

Current (at mV)	LFD (non-obese) (pA/pF)	*n*	HFD (obese) (pA/pF)	*n*	*p*
*I_K_* at +100	23.52 ± 1.32	10	50.7 ± 3.45^*^	9	0.00002
*I_Kur_* at +100	12.71 ± 0.74	18	13.36 ± 0.78	9	0.56
*I_K1_* at −120	−27.25 ± 1.67	20	−26.3 ± 2.09	11	0.73
*I_Ca,L_* at +10	−7.88 ± 0.57	16	−2.40 ± 0.68^*^	5	0.00010

*Data are means ± S.E.M*.

* *p < 0.05 compared to LFD non-obese controls, one-way ANOVA and Bonferroni test*.

Next, we assessed whether currents generated by KCNA5 and KCNJ5 channel subunits are sensitive to HFD feeding. We measured *I_Kur_* (KCNA5) and *I_K1_* (KCNJ5) in atrial myocytes. Figure 1D shows *I_Kur_* current measured in atrial myocytes isolated from LFD-fed guinea pig atria. As demonstrated in Figure 1E, *I_Kur_* density measured in HFD atria myocytes was not different (*p* > 0.05) from control at all potentials tested (Figure 1F). At +100 mV, *I_Kur_* density was 12.71 ± 0.74 pA/pF (*n* = 18, Figure 1F) and 13.36 ± 0.78 pA/pF (*n* = 9, Figure 1F, see Table 2) for LFD and HFD, respectively.

A similar picture emerged with *I_K1_* (Figure 1G); at −120 mV *I_K1_* density was −27.25 ± 1.67 pA/pF (*n* = 20) and −26.3 ± 2.09 pA/pF (*n* = 11, Table 2) in LFD and HFD atrial myocytes, respectively (Figure 1I). This finding suggests that HFD feeding does not indiscriminately affect all potassium currents.

We next determined whether *I_Ca,L_* density is affected in response to HFD feeding. Compared to LFD-fed control atrial myocytes (Figure 1J), HFD-fed guinea pigs displayed a significantly blunted *I_Ca,L_* density (Figure 1K). Atrial myocytes from HFD-fed guinea pigs displayed a significantly reduced *I_Ca,L_* peak density (at +10 mV) compared with LFD-fed controls (reduced by 69.5% or from −7.88 ± 0.57 pA/pF in LFD (*n* = 16, Table 2) vs. −2.40 ± 0.68 pA/pF in HFD (*n* = 5); ^*^p < 0.05, Figure 1L). The pooled current-voltage (*I-V*) relationship further revealed a depolarization shift (10 mV) in HFD atrial cells (Figure 1L) and supported previous reports (Gaspo et al., 1997; Mancarella et al., 2008; Nattel and Dobrev, 2017) that depression of *I_Ca,L_* contributes to atrial cell dysfunction leading to vulnerability to AF.

### Effects of High-Fat Diet on the Electrical Activity of Atrial Myocytes

**Figure 2:**
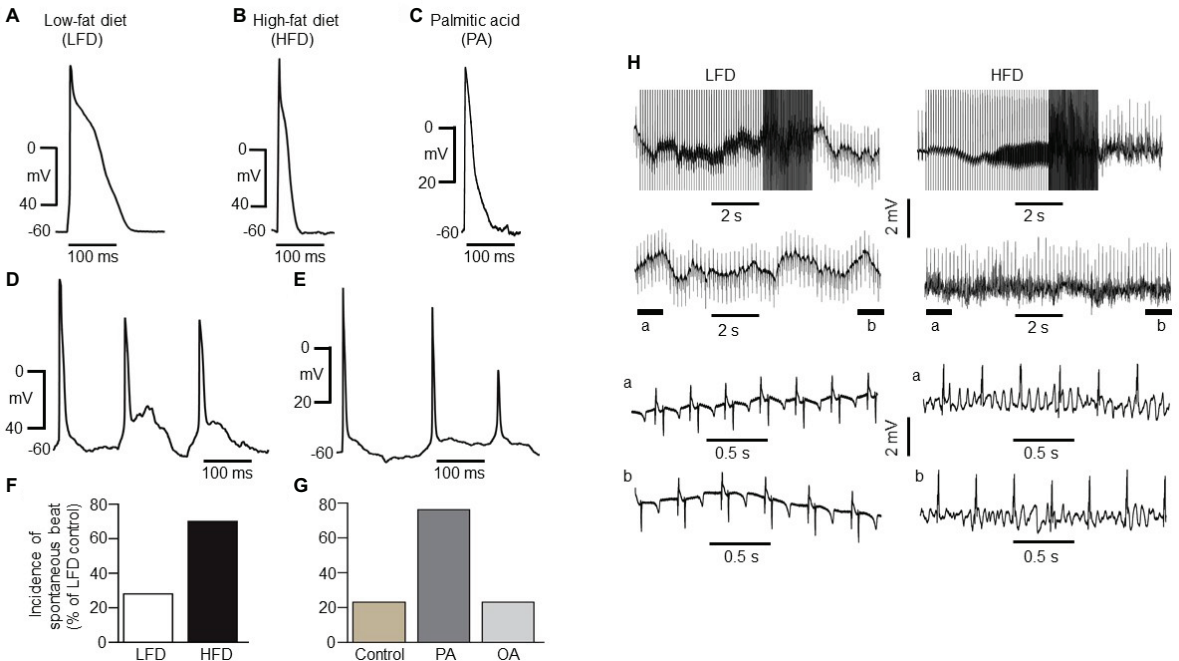
Electrical properties of guinea pig atrial myocytes and *ex vivo* hearts. **(A)** Examples of AP waveforms measured in right atrial myocytes isolated from LFD- **(A)** or HFD-fed **(B)** guinea pigs, or exposed to exogenous PA (1 mM, ≥1 h, **C**). HFD and PA pre-treated myocytes had a shortened action potential in line with the previous finding (Aromolaran et al., 2016). **(D)** Typical spontaneous beats with early delayed afterdepolarizations (EAD)-like activity measured in right atrial myocytes from HFD-fed guinea pigs. **(E)** Exemplar delayed repolarization with irregular and spontaneous beats in atrial myocytes pretreated with PA. Compared to control myocytes isolated from LFD-fed guinea pigs (**F**, white column), untreated (**G**, gold column) or pre-treated with OA (**G**, light gray column), the incidence of spontaneous beats is significantly greater in HFD (**F**, black column) and PA-treated myocytes (**G**, dark gray column). **(H)** Induction of arrhythmogenesis in right atria of isolated Langendorff perfused hearts from LFD- (left panel) and HFD-fed (right panel) guinea pigs. With rapid right atrial pacing (burst, rectangular pulses 10 ms, 1 V in amplitude with pulse-pulse duration ranging between 20 and 250 ms). Expanded view of atria electrical activities revealed sinus rhythm with LFD (left panel, bottom traces) and uncoordinated atrial activities in HFD-fed guinea pigs (right panel, bottom traces) consistent with AF. Data were generated from six different guinea pig cardiomyocyte preparations.

To examine the electrical activity of HFD-fed atrial myocytes, we measured atrial action potential, because the dysfunction of *I_K_* (or *I_Kr_* and *I_Ks_*) (Caballero et al., 2010; Gonzalez de la Fuente et al., 2013; Aromolaran et al., 2014; Puckerin et al., 2016) and *I_Ca,L_* contribute to arrhythmias (Van Wagoner et al., 1999; Mancarella et al., 2008; Barana et al., 2014), and might trigger arrhythmogenic events in HFD. We used current clamp electrophysiology to assess atrial electrical activity. Figure 2A shows action potential waveforms measured in freshly isolated atrial myocytes obtained from LFD-fed hearts. With HFD feeding (Figure 2B) and exogenous pretreatment (1 h) with palmitic acid (PA) (1 mM, Figure 2C), right atrial myocytes APD was abbreviated in line with our previous findings (Aromolaran et al., 2016). Furthermore, HFD atrial myocytes displayed irregular spontaneous beats with early-afterdepolarization (EAD)-like activity (Figure 2D). Similarly, PA-treated myocytes displayed delayed repolarization with irregular and spontaneous beats (Figure 2E).

HFD atrial myocytes [70% or 21 out of 30 cells vs. 6 out of 23 cells (or 26%) for LFD controls, Figure 2F] or PA-treated myocytes [76% or 22 out of 29 cells vs. 3 out of 13 cells (or 26%) for untreated atrial myocytes, Figure 2G] revealed a higher incidence of spontaneous beats. By contrast, 3 out of 15 cells (or 20%) pre-exposed to oleic acid (OA, 1 mM) were arrhythmogenic (Figure 2G). Thus, our data in addition to the findings of Aromolaran and others (Aromolaran et al., 2016) confirm that HFD and PA are pro-arrhythmic while OA is anti-arrhythmic in guinea pigs.

To determine whether HFD hearts will be vulnerable to AF/tachycardia, burst stimulation and extrasystolic beats combined (Mancarella et al., 2008; Diness et al., 2010; Osadchii, 2012; Ashrafi et al., 2016) were delivered in the atria (Mancarella et al., 2008; Diness et al., 2010; Ashrafi et al., 2016) of 50-day-old isolated Langendorff-perfused hearts (Mancarella et al., 2008). We have defined induction of AF as rapid, irregular atrial activity lasting ≥2 s. Compared to LFD-fed hearts (three out of three, Figure 2H, left panel), AF/tachycardia was readily inducible in HFD-fed hearts (three out of three, Figure 2H, right panel), following application of burst stimulation protocol (10 ms and 1 V rectangular pulses, with pulse-pulse durations ranging between 20 and 250 ms). Together, our data demonstrate that increased *I_K_* and decreased *I_Ca,L_* densities predispose to atrial arrhythmogenesis and AF/tachycardia in HFD-fed guinea pigs.

### Patch-Clamp Recordings From Atrial Myocytes After Stimulation of the Atrium

**Figure 3:**
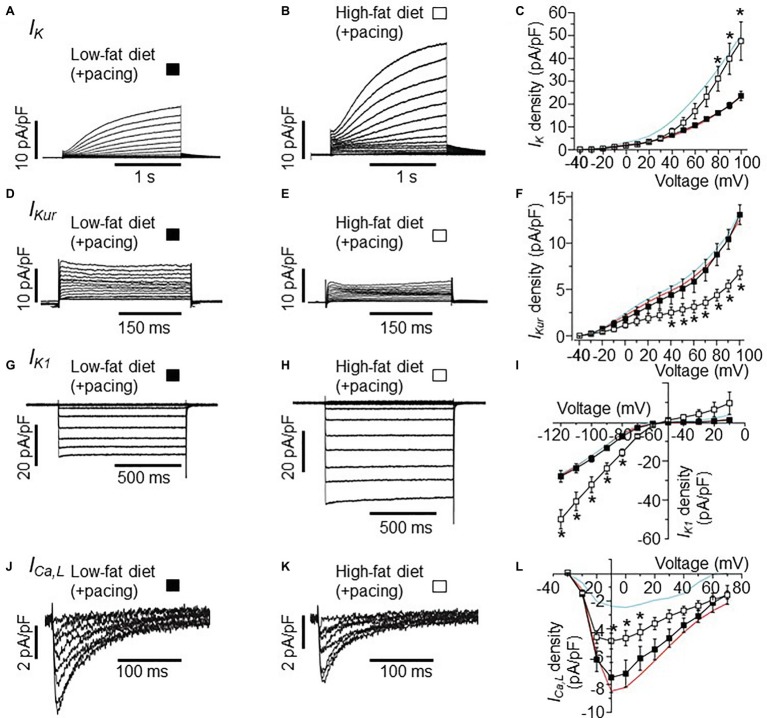
Effects of burst pacing on membrane currents in cardiomyocytes isolated from the atria of LFD and HFD guinea pigs. **(A)** Selected *I_K_* tracings of myocytes from LFD atria after burst pacing. **(B)** *I_K_* currents measured in *HFD*-fed guinea pigs after burst pacing. **(C)** Pooled *I-V* plots for *I_K_* density in LFD (■, *n* = 10) and HFD (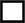, *n* = 5) myocytes. Data for *I_K_* measured without atria pacing is reproduced for LFD (red trace) and HFD (cyan trace) from Figure 1C. **(D–L)** Exemplar currents and *I*-*V* curves for *I_Kur_*, *I_K1_*, and *I_Ca,L_*, same format as **(A–C)**. *I_Kur_* density is reduced **(F)** after pacing while *I_K1_* density is increased **(I)**. Each data point represents mean ± S.E.M, *n* = 5–20. ^*^p < 0.05. Data were generated from six different guinea pig cardiomyocyte preparations.

Next, we tested the hypothesis that the combination of HFD and pacing might result in further remodeling of the expression of atrial ion channels. Therefore, we studied the electrophysiological properties of *I_K_*, *I_Kur_*, *I_K1_*, and *I_Ca,L_* in atrial myocytes isolated from LFD and HFD-fed guinea pigs after pacing. Whole-cell currents were measured in freshly isolated atrial myocytes within 2–6 h. after stimulations of the atrium (burst stimulation and extrasystolic beats combined). Figures 3A,B shows *I_K_* current traces recorded in LFD and HFD atrial myocytes that were subjected to pacing. *I*-*V* curves revealed that *I_K_* currents remained significantly larger (101.5% increase at +100 mV), when compared to LFD control *I_K_* currents (Figure 3C). At +100 mV, averaged *I_K_* densities were 23.52 ± 1.32 pA/pF (*n* = 10) and 47.4 ± 8.38 pA/pF for LFD and HFD (^*^p < 0.05, Table 3), respectively (Figure 3C). By contrast, we found that *I_Kur_* and *I_K1_* densities measured in HFD-fed atrial myocytes were altered with pacing. Figure 3D shows control *I_Kur_* currents measured in LFD atrial myocytes. As illustrated in Figure 3E, *I_Kur_* density is severely depressed in HFD myocytes subjected to pacing. At +100 mV, *I_Kur_* density was reduced from 12.71 ± 0.74 pA/pF (*n* = 18) to 6.80 ± 0.72 pA/pF (*n* = 6, ^*^p < 0.05) (or by ~49%, Figure 3F; Table 3).

**Table 3:** Effects of atrial burst pacing on averaged ion current densities measured in myocytes isolated from non-obese and obese guinea pigs after 50 days.

Current (at mV)	LFD (non-obese) (pA/pF)	*n*	HFD (obese) (pA/pF)	*n*	*p*
*I_K_* at +100	23.52 ± 1.32	10	47.4 ± 8.38^*^	5	0.001
*I_Kur_* at +100	12.71 ± 0.74	18	6.80 ± 0.72^*^^,^^#^	6	0.000032
*I_K1_* at −120	−27.25 ± 1.67	20	−49.72 ± 4.95^*^	11	0.0016
*I_Ca,L_* at +10	−7.88 ± 0.57	16	−4.70 ± 0.68^*^	5	0.0014

*Data are means ± S.E.M*.

* *p < 0.05 compared to LFD non-obese controls, one-way ANOVA and Bonferroni test*.

# *p < 0.05 compared to corresponding HFD obese without pacing, one-way ANOVA, two-tailed unpaired t test*.

Under similar conditions, *I_K1_* density (Figure 3G) was increased from −27.25 ± 1.67 pA/pF (*n* = 20) to −49.72 ± 4.95 pA/pF (Figure 3H, or by 82.4%, ^*^p < 0.05) at −120 mV with HFD and pacing (Figure 3I). With *I_Ca,L_*, the depression in current seen in HFD was not as pronounced with pacing such that the *I_Ca,L_* density measured at +10 mV in HFD (without pacing) was increased from −2.40 ± 0.68 pA/pF (*n* = 7) to −4.70 ± 0.68 pA/pF (*n* = 5, Figures 3J–L; Tables 2, 3) with pacing. Our data suggest that further remodeling of *I_Kur_* and *I_Ca,L_* seen with HFD and atrial pacing may be compensatory mechanisms to account for the profound changes in *I_K_* and *I_K1_* densities.

### Effects of High-Fat Diet and Pacing on the Biophysical Properties of *I_K_*

**Figure 4:**
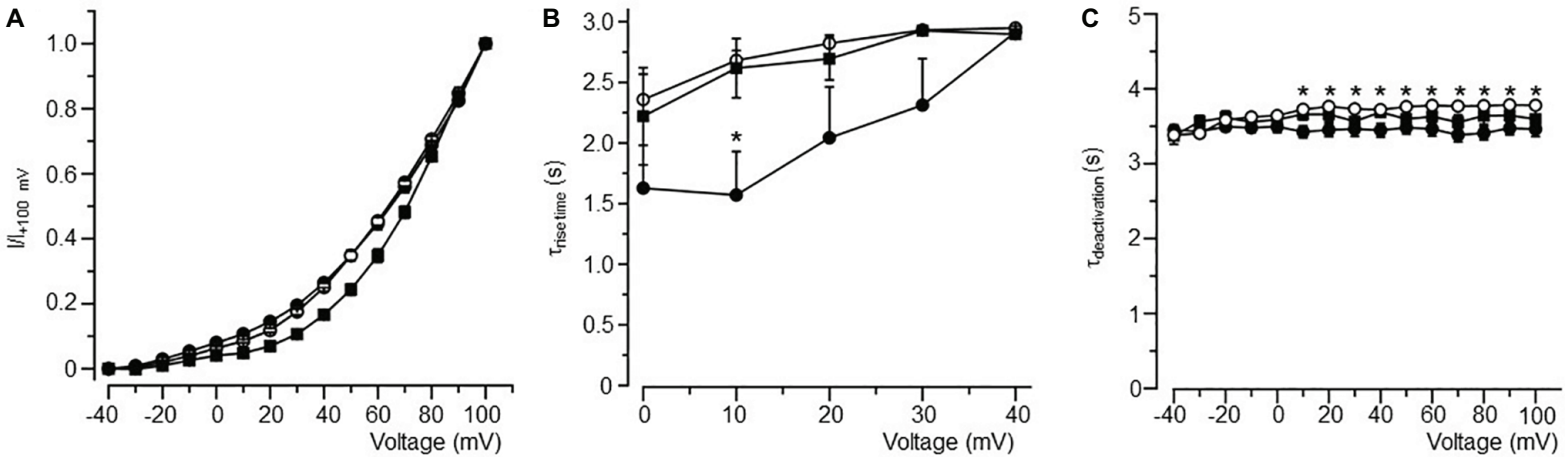
Biophysical properties of *I_K_* in atrial myocytes isolated from LFD- and HFD-fed (with and without burst pacing) guinea pigs. **(A)** Normalized activation curves for *I_K_* in LFD (●, *n* = 10), HFD (○, *n* = 9), and HFD with pacing (■, *n* = 5). **(B)** Relationship between time course (*τ*_rise time_) of activation and voltage. **(C)** Plot of the *I_K_* tail current deactivation (*τ*_deactivation_) relative to voltage. Gating properties of *I_K_* are altered with HFD alone or in combination with pacing. ^*^p < 0.05 relative to LFD control myocytes. Unpaired Student’s *t*-test followed by one-way ANOVA.

The ability of HFD to increase *I_K_* was of particular interest given that an increase in *I_Ks_* is an important signature of AF (Caballero et al., 2010; Gonzalez de la Fuente et al., 2013; Perez-Hernandez et al., 2016). Therefore, we assessed the biophysical properties (Figure 4) of *I_K_* in LFD, HFD, and HFD (with pacing) atrial myocytes. The voltage-dependence of activation of *I_K_*, obtained by normalizing the activation curves to the peak current at +100 mV, was not altered in HFD alone myocytes when compared to LFD control myocytes but displayed a slight rightward shift in activation in HFD myocytes subjected to pacing (Figure 4A).

*I_K_* channels had a slower rise time of activation (*τ*_rise_, Figure 4B) and deactivation (*τ*_deactivation_, Figure 4C) kinetics in HFD myocytes. Averaged *τ*_rise_ was 1.57 ± 0.36 s (*n* = 10) and 2.68 ± 0.08 s (*n* = 9, ^*^p < 0.05, Table 4), while *τ*_deactivation_ was 3.43 ± 0.08 s (*n* = 10) and 3.72 ± 0.03 s (*n* = 10, ^*^p < 0.05, Table 4) at +10 mV for LFD and HFD myocytes, respectively. With pacing, *τ*_rise_ is unchanged while *τ*_deactivation_ is slightly faster (HFD 3.72 ± 0.03 s vs. HFD with pacing 3.65 ± 0.07 s, Figure 4C; Table 4).

**Table 4:** Time constants for current activation (*τ*_rise_) and deactivation (*τ*_deactivation_) for *I_K_* channels measured in atrial myocytes isolated from LFD non-obese and HFD obese guinea pigs (with and without pacing) after 50 days.

Experimental condition	*τ*_rise_ (s) at +10 mV	*p*	*τ*_deactivation_ (s) at +10 mV	*p*	*n*
LFD non-obese	1.57 ± 0.36		3.43 ± 0.08		10
HFD obese	2.68 ± 0.08^*^	0.01075	3.72 ± 0.03^*^	0.00431	9
HFD obese (+atrial pacing)	2.61 ± 0.24^*^	0.0321	3.65 ± 0.07	0.08777	5

*Data are means ± S.E.M*.

* *p < 0.05 compared to LFD non-obese controls, one-way ANOVA and Bonferroni test*.

**Figure 5:**
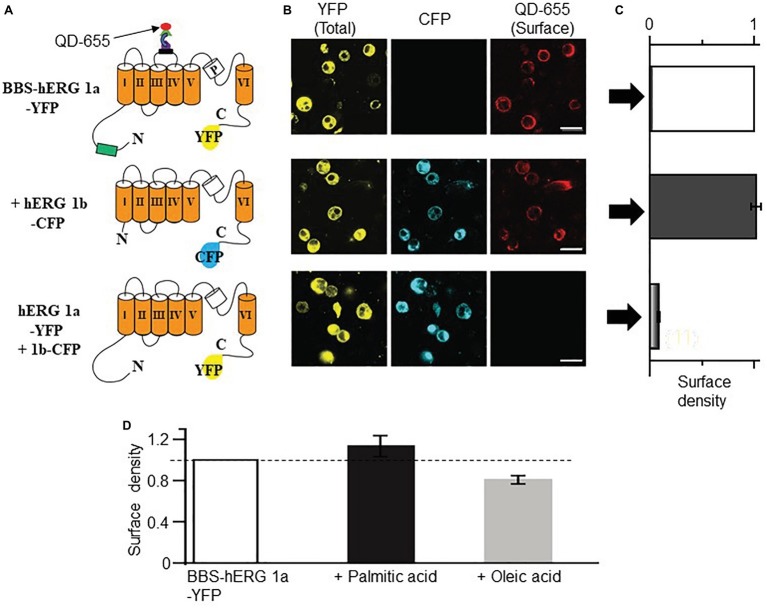
Optical detection of surface hERG subunits with quantum dot in HEK293 cells. **(A)** Schematic of BBS- and YFP-tagged hERG 1a, CFP-tagged hERG 1b and YFP-tagged hERG 1a lacking the BBS tag. **(B)** Confocal images of non-permeabilized HEK293 cells expressing BBS-hERG 1a-YFP or hERG 1a-YFP alone and in combination with hERG 1b-CFP, and exposed to QD_655_ to selectively label surface channels. Panels show hERG channels for YFP (total expression, left panel), CFP (middle panel), and QD_655_ (surface channels, right panel). Scale bars represent 20 μm. **(C)** Normalized mean QD_655_ signals from YFP-positive cells expressing BBS-hERG 1a-YFP alone (white column), BBS-hERG 1a-YFP/hERG 1b-CFP (black column), and hERG 1a-YFP/hERG 1b-CFP (gray column) represent relative surface density. hERG 1a-YFP/hERG 1b-CFP serves as negative control and shows negligible QD_655_ signal consistent with lack of BBS-tag and therefore negligible channel surface density. **(D)** Compared to untreated HEK293 cells transiently expressing BBS-HERG 1a-YFP, pretreatment (≥1 h) with PA (1 mM) increased the surface density of hERG channels (~13%), while OA reduced (~20%, light gray column). Dotted line indicates 1. Numbers in parenthesis indicates the number of independent cell culture experiments.

The increase in *I_K_* also suggests that HFD may modulate the surface expression of *I_K_* channel subunits (KCNQ1 and hERG). We used our bungarotoxin-binding assay (BBS) developed for KCNQ1 subunits (Aromolaran et al., 2014), to test the impact of FFAs elevated in obesity (Aromolaran et al., 2016) on *I_K_* channel subunits. First, we validated the feasibility of using this optical assay to assess hERG channel subunits. Figure 5A shows cartoon representation of BBS- and YFP-tagged hERG subunits. Cell surface hERG 1a channel subunits were selectively labeled by exposing BBS-hERG 1a-YFP expressing non-permeabilized HEK293 cells to biotinylated bungarotoxin (BTX-Biotin) followed by streptavidin-conjugated quantum dot-655 (QD_655_) (Aromolaran et al., 2014). Confocal images of cells expressing BBS-hERG 1a-YFP alone or in combination with hERG 1b-CFP displayed robust red QD_655_ fluorescence labeling of the cell periphery (Figure 5B).

To quantify the QD_655_ signal, cells were subjected to flow cytometry. The mean QD_655_ fluorescence of YFP-positive cells was defined as channel surface density or extent of channel surface expression. Measured QD_655_ signals are normalized to YFP fluorescence (which corresponds to channel protein levels). Quantification of mean QD_655_ fluorescence indicated that BBS-hERG 1a-YFP alone or when combined with hERG 1b-CFP displayed similar surface density (Figure 5C, 2% increase or from surface density 1.0, *n* = 12 separate cultures, to 1.02 ± 0.05, 8 separate cultures), in line with published data, indicating hERG 1a traffics independently of hERG 1b to the cell surface in heterologous cells (Phartiyal et al., 2008). Negative controls expressing untagged hERG 1a and 1b displayed no QD_655_ fluorescence (averaged surface density was 0.18 ± 0.006, five separate cultures), confirming that the red fluorescence signal was specific and only detected the BBS tag, consistent with our previous reports on KCNQ1 trafficking in HEK293 cells and cardiomyocytes (Aromolaran et al., 2014; Kanner et al., 2018).

We used the QD_655_-labeling approach coupled with flow cytometry to assess the effects of PA (1 mM) and OA (1 mM) on hERG channel surface density as a potential mechanism for increased *I_K_* acquired in HFD. Figure 5D shows the effects of PA and OA on the surface density of hERG 1a channels. Relative to control HEK293 cells expressing BBS-hERG channel (from four separate cultures), quantification of mean QD_655_ fluorescence signal revealed that pretreatment with PA increased the surface density of hERG channels (~13 ± 1%, three separate cultures), whereas OA reduced (~20 ± 0.2%, four separate cultures) the surface density of hERG 1a channels, consistent with our previous report that PA increases hERG currents while OA reduced them in HEK293 cells (Aromolaran et al., 2016). Together, the data suggest that the increase in *I_K_* is most likely due to deficits in channel gating and increase in channel trafficking.

### Effects of High-Fat Diet and Rapid Atrial Pacing on Human Atrial Electrophysiology Models

**Figure 6:**
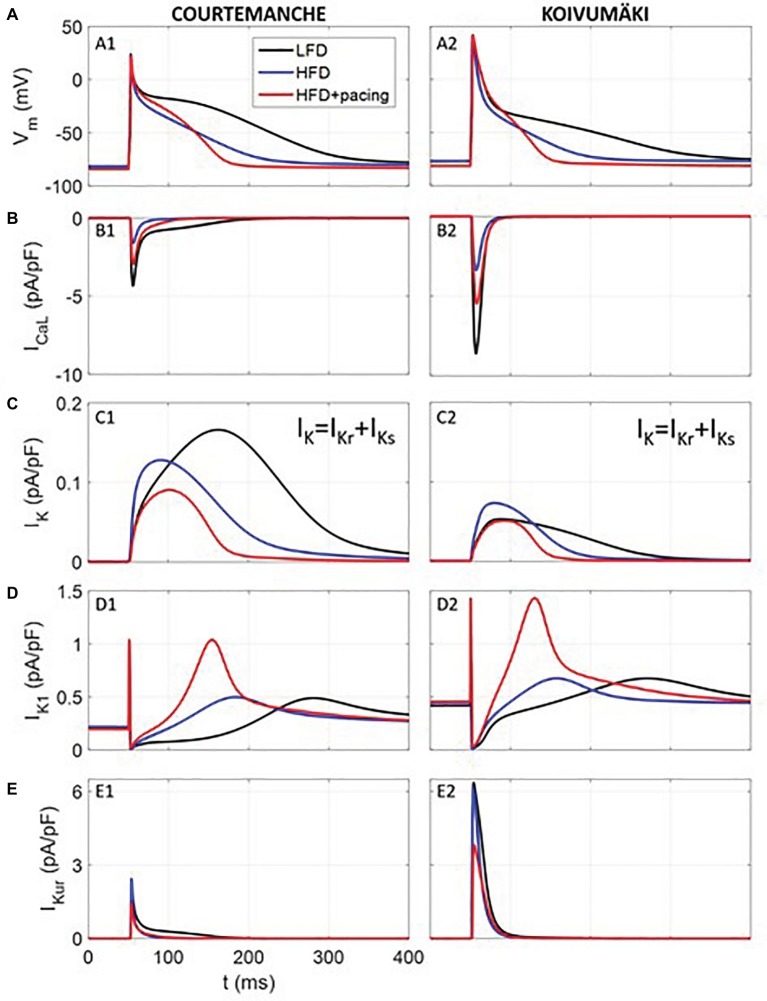
Simulated electrical activity of human atrial myocytes corresponding to LFD and HFD (with and without pacing) individuals. Action potential **(A)** and *I_Ca,L_* **(B)**, *I_K_* **(C)**, *I_K1_* **(D)**, and *I_Kur_* **(E)** current densities. Rapid atrial pacing caused more action potential shortening compared with HFD alone (blue trace), which was intermediate between LFD (black trace) and HFD + pacing (red trace).

To determine whether the effects of HFD-feeding alone or in combination with rapid atrial pacing on whole cell ionic conductance would alter human atrial electrophysiology, we utilized the Courtemanche (Courtemanche et al., 1998) and Koivumäki (Koivumäki et al., 2011) human atrial cardiomyocyte models. Computer simulation (Table 1) results are summarized in Figure 6. As depicted, our simulated human atrial APs (Figure 6A) reveal a strong APD reduction in HFD compared to LFD myocytes, which agrees with our experimental results. APD at 90% repolarization (APD_90_) was reduced approximately by ~41, ~51, and ~68% in simulated human APs from Courtemanche and Koivumäki models and experimentally recorded guinea pig APs, respectively.

As observed in Figure 6, where traces of current densities are shown in panels B-E (*I_K_* was composed of *I_Kr_* and *I_Ks_*), APD_90_ reduction in APs from HFD individuals was mainly due to *I_Ca,L_* current reduction in the first 50 ms of the AP. In fact, when only considering the *I_Ca,L_* downregulation, APD_90_ was reduced by 32 and 29% for Courtemanche and Koivumäki models, respectively, which means that the effect of the *I_Ca,L_* decrease was stronger than the effect of the *I_K_* increase in the APD_90_ shortening. In addition, *I_Kr_* had a stronger effect than *I_Ks_* in the APD_90_ reduction since when considering the *I_Ca,L_* downregulation together with the *I_Kr_* upregulation, APD_90_ shortened by 39% (Courtemanche) and 48% (Koivumäki). In our paced HFD myocytes simulations, APD_90_ decreased by 55% (Courtemanche) and 62% (Koivumäki) for paced HFD myocytes compared to LFD (by 24% (Courtemanche) and 21% (Koivumäki) if compared to HFD without pacing). As observed in Figure 6, APD_90_ reduction was due to the downregulated *I_Ca,L_* and upregulated *I_K1_* currents (when only considering those two currents variations, the APD_90_ shortened by 60 and 80% for the Courtemanche and Koivumäki models, respectively), while downregulation of *I_Kur_* current prolonged the APD_90_. The effect of the *I_Kur_* downregulation on the APD_90_ prolongation seems to be more intense for the Koivumäki model. In addition, the maximum AP amplitude of the simulated APs with the Courtemanche and Koivumäki models did not vary significantly in HFD compared to LFD myocytes (5% of increase for experiments), while it did increase by 3% after pacing.

## Discussion

In this study, we show that there is a surprising heterogeneity of distinct atrial potassium currents that are sensitive to HFD and underlie the increased susceptibility of obese heart to atrial arrhythmogenic events. Our data further demonstrate that HFD discriminately increases *I_K_* density through posttranslational (protein trafficking and channel gating defects) modulation of channel subunits. Only when combined with burst atrial pacing did the altered functional expression of *I_Kur_* and *I_K1_* phenotypes emerge, manifested as severely reduced *I_Kur_* density, and a marked increase in *I_K1_* current density. To our knowledge, this is the first study showing that the electrical abnormalities in HFD-fed guinea pig atria included a decrease in *I_Ca,L_* density. Therefore, we conclude that both increased *I_K_* and reduced *I_Ca,L_* functional expressions are key events that underlie the initial HFD-mediated atrial electrical remodeling and arrhythmogenesis with implications for atrial fibrillation.

### Comparison to Previous Studies on High-Fat Diet, K Channels, and Cardiac Electrical Remodeling

In heart, increased *I_K_* density (Caballero et al., 2010; Gonzalez de la Fuente et al., 2013; Aromolaran et al., 2016) is an important signature of atrial electrical remodeling and is therefore critically associated with abbreviation of APD and supraventricular arrhythmias (Nattel and Dobrev, 2017). Understanding how HFD affects the functional properties of *I_K_* is likely to inform our knowledge regarding vulnerability to atrial arrhythmogenesis in obese patients.

With the exception of our previous report (Aromolaran et al., 2016) on the effect of HFD-induced obesity on *I_K_* functional properties, we are not aware of other studies that have assessed modulation by HFD of *I_K_* function. Our current findings confirm our previous observations (Aromolaran et al., 2016) and also show that in atrial myocytes from HFD-fed guinea pigs, *I_K_* density was approximately two-fold higher, the rise time of activation and deactivation kinetics were slower, and the conductance curve was essentially unchanged when compared with LFD-fed control myocytes.

To further elucidate the molecular mechanisms underlying HFD-mediated effects on *I_K_*, we assessed the effects of saturated PA and the monounsaturated OA on the trafficking properties of hERG channel subunits. Previously, we reported that PA likely mediates the effects of obesity on *I_K_* in atrial myocytes. In the present study, we discovered that PA increased the surface expression of hERG channel subunits consistent with the notion that the facilitatory effect of HFD on *I_K_* involves multiple signaling pathways. These findings may inform experimental strategies for predicting the effects of HFD mechanisms on atrial electrical and cardiac function. Together, our findings provide the new insight that the facilitatory effect of HFD on *I_K_* can be attributed to channel gating and trafficking defects.

There have been conflicting data regarding modulation by HFD of atrial specific *I_Kur_* (or K_v_1.5 encoded by KCNA5) (Bhuyan and Seal, 2017) functional expression. Yifan and colleagues (Zhang et al., 2016), demonstrated that the protein expression levels of K_v_1.5 was significantly increased in atria of obese mice after 8 weeks. By contrast, Morrow and others (Huang et al., 2013) reported reduced mRNA and protein levels of ventricular K_v_1.5 channel subunits (Huang et al., 2013) in HFD-induced obese mice after 20 weeks. The changes reported by Morrow’s group were also associated with ventricular electrical remodeling (including impaired repolarization and QT prolongation) (Huang et al., 2013). Whether changes in K_v_1.5 expression translates to altered *I_Kur_* current density is unknown since electrophysiology measurements were not performed in these studies. Therefore, a definitive role for altered *I_Kur_* density in HFD-induced cardiac electrical dysfunction requires further investigation.

The differences between our study and these studies may be due to species differences (guinea pig vs. mice) (Zhang et al., 2016), differences in cell-type (atria vs. ventricles) (Huang et al., 2013) or duration of HFD or HFD-induced obesity (7 vs. 20 weeks). Future studies will be needed to further understand the effects of HFD on the spatial selectivity and temporal properties of K_v_1.5 transcript and protein levels.

There is currently a lack of clarity about the effects of HFD on *I_K1_* functional expression and atrial arrhythmogenesis. In a HFD obese rat model, Boyet and others (Ashrafi et al., 2016) demonstrated that mRNA levels of Kir2.1 (KCNJ2) and *I_K1_* current density are significantly increased after 8 weeks. However, these changes had negligible effects on ventricular APD (Ashrafi et al., 2016). Similar to *I_Kur_*, *I_K1_* density is also unchanged in our studies, demonstrating that *I_Kur_* and *I_K1_* channels are insensitive to HFD in guinea pig atrial myocytes.

With the sole exception of *I_K_*, our data revealed that *I_Kur_* and *I_K1_* current densities are not affected by HFD. This finding is in contrast to the results of Boutjdir and others (Mancarella et al., 2008), which shows that *I_K_* density is not altered in an α-1D KO mice model of AF. A similar reduction of *I_Ca,L_* density with no compensatory changes from other currents has also been described in sinoatrial node cells of the same α-1D calcium channel KO mice (Mangoni et al., 2003). One can speculate that the molecular mechanisms that underlie altered function of distinct atrial ion channels leading to supraventricular arrhythmias may differ depending on the underlying pathology.

### Effects of Rapid Pacing on the Atrial Electrophysiology of High-Fat Diet-Fed Guinea Pig

**Figure 7:**
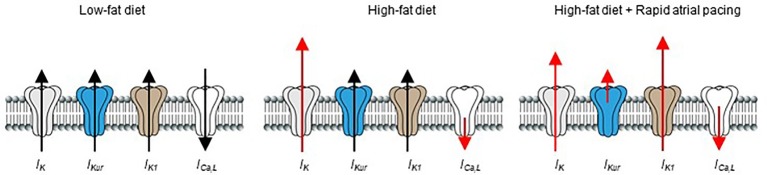
Schematic representation of the effects of HFD and rapid pacing on atrial electrical properties in guinea pig heart. Short-term high-fat diet induced significant electrical remodeling of *I_K_* and *I_Ca,L_* while *I_Kur_* and *I_K1_* remained unchanged compared to LFD controls. When HFD is combined with pacing *I_Kur_* is significantly reduced while *I_K1_* is increased in line with their roles in pathogenesis of supraventricular arrhythmias such as atrial fibrillation. The size of red arrows indicates change in the current densities of the ion channels.

Previous reports have shown that rapid pacing (acute vs. chronic) cause atrial electrophysiological remodeling in humans (Goette et al., 1996; Daoud et al., 1997; Yue et al., 1999; Hadian et al., 2002), and in different animal models of AF (Yu et al., 1998; Tsuchiya et al., 2009; Laszlo et al., 2011). Our data show that acute or short-term rapid pacing increases vulnerability of HFD-fed guinea pigs to atrial arrhythmogenesis compared to LFD-fed controls. Our results also showed that, while the functional expression of *I_K_* (increased) and *I_Ca,L_* (decreased) were essentially unchanged after rapid pacing; *I_Kur_* density was severely depressed and *I_K1_* current density was increased, in agreement the ability of a brief (10–15 min) episode of rapid atrial pacing to induce cardiac electrical remodeling (Figure 7; Yu et al., 1998; Hadian et al., 2002).

Regarding pacing-induced downregulation of *I_Kur_* and upregulation of *I_K1_* densities, findings have been contradictory. In agreement with our findings, *I_Kur_* amplitude is significantly reduced in human atrial myocytes at rapid activation rates (Feng et al., 1998; Ehrlich et al., 2008). By contrast, Nattel and colleagues (Yue et al., 1999) did not find any significant changes in the densities of *I_Kur_* and *I_K1_* in their study. *I_K1_* is also not affected in a rabbit model of rapid atrial pacing (RAP) (Laszlo et al., 2007). In our studies, we utilized 300 beats/min, compared to 400 beats/min and 600 beats/min used in these studies of Nattel and colleagues (Yue et al., 1999) and Bosch’s group (Laszlo et al., 2007). The lower rate in our model combined with a HFD genetic background might cause faster changes in current densities, consistent with the notion that the metabolic state of atria may be an important determinant of the temporal properties and/or sensitivity to pacing.

Alternatively, the different findings may also be due to interspecies differences in the time course of electrical remodeling as previously demonstrated in goat and dog RAP models of AF (Yue et al., 1997, 1999). It was demonstrated that atrial electrical remodeling occurred faster in goat when compared to dog RAP model. Electrical remodeling in a horse model has also been shown to be slower than that in goat or dog (Ausma et al., 2002).

It is reasonable to speculate that the depression of *I_Kur_* density due to rapid pacing would be expected to prolong APD, and therefore may represent a compensatory mechanism to limit further electrical remodeling and prevent onset of atrial arrhythmogenic events. Our data further suggest that increased *I_K1_* density would be expected to play a critical role in promoting electrical remodeling and atrial arrhythmogenesis leading to the maintenance of supraventricular arrhythmias.

Concerning the extrapolation of our results to humans through computational modeling and simulations, our findings show that remodeling due to obesity might also have proarrhythmic effects. The combination of increased *I_K_* and reduced *I_Ca,L_* in HFD myocytes accelerates repolarization and leads to shorter APDs and shorter refractory period that favor arrhythmogenesis (ectopic and/or reentrant activity). Furthermore, our data suggest that AF mechanisms including the strong APD_90_ reduction coupled with EAD-like activity and delayed repolarization with irregular and spontaneous beats would explain the higher predisposition of obese individuals to develop atrial arrhythmias (Wanahita et al., 2008). Simulations also suggest that rapid pacing increases vulnerability of obese individuals to arrhythmias, possibly by exacerbating AF mechanisms. This suggestion is in accordance with our experimental results showing that APD_90_ is shorter with pacing compared to without pacing.

Altogether, we can surmise that in our case, the current variations in humans were similar to those observed in the guinea pig experiments; therefore, the effect of obesity in humans would have a similar behavior to our guinea pig model, as demonstrated by our computer simulations. However, further experiments with human atrial myocytes are needed to validate if variations in the ionic currents are comparable to the ones observed in guinea pig atrial cells.

### Study Limitations

This study focused entirely on understanding the effects of HFD feeding on ion channel mechanisms that either initiate or maintain supraventricular arrhythmias. A major limitation of the study is that we did not model the effects of PA and OA on atrial APD, or susceptibility of HFD myocytes to develop EADs, DADs, and spontaneous depolarizations. Our current data, in addition to our previous reports (Aromolaran et al., 2016), strongly suggest that PA will mimic the effects of HFD, while OA will be expected to prevent arrhythmogenic AF mechanisms.

Although our results revealed a significant downregulation of global atrial *I_Ca,L_* in HFD-fed guinea pigs, it does not differentiate between a role for α-1C and α-1D channels both of which contribute prominently to total *I_Ca,L_* (Barana et al., 2014). The effects of HFD on the functional expression of *I_Ca,L_* have been investigated in rat models but with contrasting outcomes. For example, in rats fed a high-fat diet for 15 weeks, it was found that the gene and protein expressions of α-1C calcium channel were unchanged (Lima-Leopoldo et al., 2008; Leopoldo et al., 2011). However, in an unrelated study by the same authors, mRNA expression of α-1C calcium channel is increased at 30 weeks (Lima-Leopoldo et al., 2013). Boyett and others (Ashrafi et al., 2016) also reported increased mRNA levels of ventricular *I_Ca,L_* after 8 weeks in high fat diet fed rats; while Leopoldo and others (Leopoldo et al., 2011) found no change in protein expression after 15 weeks. In a diet-induced obese *Psammomys obesus* Gerbil model, mRNA and protein expression levels of the α-1C calcium channel were decreased after 16 weeks (Sahraoui et al., 2016). Furthermore, Gaborit and others (Gaborit et al., 2005) have also shown a significant reduction in the gene expression of α-1C/α-1D in AF patients suggesting a role of both channels in the pathogenesis of AF. Whether or how the functional expression of α-1C and α-1D calcium channels is altered in obese or lipotoxic atria and dyslipidemia is unknown. A previous report by the Boutjdir group (Mancarella et al., 2008) showed that while α-1C mRNA levels were unchanged in α-1D KO mice of AF, *I_Ca,L_* current density is significantly reduced supporting a role for the α-1D calcium channel in AF. It is intriguing to speculate that similar molecular mechanisms of differential α-1C and α-1D calcium channel expression may underlie the severe reduction of *I_Ca,L_* and propensity for atrial arrhythmogenesis observed in our model.

Other mechanisms such as a reduced intracellular calcium transient amplitude (Mancarella et al., 2008) may have also contributed to the adverse changes in *I_Ca,L_*. Therefore, studies that distinguish between the functional expression of Ca_v_1.2/Ca_v_1.3, and altered calcium handling proteins (Aromolaran and Boutjdir, 2017), with implications for reduced *I_Ca,L_* are likely to provide critical insights for devising targeted therapies in obese patients.

Another limitation of this study is that we did not assess the effects of HFD feeding on the separate components (*I_Kr_* and *I_Ks_*) of *I_K_*. Differential modulation of *I_Kr_* and *I_Ks_* in response to drugs and neurotransmitters has been proposed. For example, Sanguinetti and Jurkiewicz (1991) demonstrated selective inhibition of *I_Kr_* by the class III antiarrhythmic drug E-4031, while the β-adrenergic agonist isoproterenol facilitated *I_Ks_* with significant effects on *I_Kr_* measured in guinea pig ventricular myocytes (Sanguinetti and Jurkiewicz, 1991; Sanguinetti et al., 1991). Furthermore, Matsuura and Ehara (1997) also provided evidence that extracellular ATP discriminately potentiated *I_Ks_* current density. Therefore, in future studies, it will be important to investigate the selective role of *I_Kr_* and *I_Ks_* in obesity-related adverse atrial electrical remodeling that promote reentry mechanisms, incidence of triggered activity, and EADs. Our studies can also be further improved by incorporating comprehensive and robust biophysical analyses (gating mechanisms including activation, inactivation, and deactivation) of remodeled currents (*I_Kr_*, *I_Ks_*, *I_Kur_*, *I_K1_*, and *I_Ca,L_*) investigated in our study and how the pattern of our results may be related to changes in resting membrane potential and contour of the final repolarization phase of the atrial action potential. Work to advance these approaches are currently been conducted in our laboratory.

Although our findings show defects in trafficking and gating mechanisms of *I_K_* may underlie adverse remodeling by obesity they do not exclude other mechanisms including increased sympathetic regulation of *I_K_* and abnormal transcriptional and posttranslational modifications. It will also be interesting in future studies to examine the contribution of these mechanisms.

Furthermore, the electrophysiological and pharmacological characteristics of guinea pig and human atrial myocytes might substantially differ and therefore, it is important to be cautious when extrapolating results from animal experiments to humans, due to the differences among species (O’Hara and Rudy, 2012). On the other hand, it is well known that the delayed rectifier current in human myocytes, as in other species, is composed of *I_Kr_* and *I_Ks_* (Wang et al., 1994). Atrial models available in the literature incorporate the two components and the vast majority present larger *I_Kr_* than *I_Ks_* currents. In fact, maximum *I_Ks_* amplitude is the smallest among all the ionic currents except for the Nygren model (Wilhelms et al., 2012). And even though the maximum *I_Ks_* current amplitude in the Courtemanche model is larger than in the Koivumäki model, upregulation of this current in the HFD yielded a similar effect in both models. Our data further show that APD_90_ shortening was mainly due to the contribution of the downregulated *I_Ca,L_* and upregulated *I_Kr_*, while *I_Ks_* slightly affected the APD_90_. Our findings also suggest that evaluating the obesity-related adverse atrial electrical remodeling observed in the present study at physiological temperatures (37°C versus 20–25°C) may be better predictive of arrhythmogenesis in patients.

## Conclusion

Taken altogether, our study is the first to show that HFD negatively modulates *I_K_* and *I_Ca,L_*. Our findings are consistent with the notion that individuals with metabolic disorders may display increased *I_K_* and reduced *I_Ca,L_* with serious implications for cardiac repolarization. Our results predict that: (1) inhibitors of hERG/KCNQ1 channels (Nakaya et al., 2000); (2) activators of Ca channel; or (3) cellular mediators that either limit *I_Kr_*/*I_Ks_* channel opening or promote *I_Ca,L_* opening would be expected to limit abbreviation of atrial APD and therefore vulnerability to supraventricular arrhythmias in patients with metabolic disorders.

By describing the first measurements of the effect of HFD on *I_K_*/*I_Ca,L_* channel function, we reveal that *I_K_*/*I_Ca,L_* channels are sensitive to pathological changes in metabolic substrates and may be involved in the initiation of supraventricular arrhythmias, since both increased *I_K_* density and downregulation of *I_Ca,L_* makes HFD-fed guinea pigs prone to supraventricular arrhythmias. In contrast to observations of increased *I_K_* or reduced *I_Ca,L_* current densities due to remodeling after atrial arrhythmias such as AF has occurred, our data are consistent with the premise that an *a priori* increase in *I_K_* (*I_Ks_*) (Caballero et al., 2010; Gonzalez de la Fuente et al., 2013) and reduction in *I_Ca,L_* (Brundel et al., 2001; Barana et al., 2014; Nattel and Harada, 2014) current density may play a *key* role in adverse atrial electrical mechanisms that underlie initiation of supraventricular arrhythmias underlain by metabolic disorders.

Furthermore, dietary interventions involving: (1) removal of dietary PA; (2) increasing dietary OA; (3) increased exercise training; and (4) multiple combinations of the different interventions may reduce arrhythmias due to obesity. In this context, a previous report by Paulino and others (Paulino et al., 2010) demonstrated that exercise and caloric restriction prevented cardiac defects in obese rats through modulation of calcium handling proteins. Recently, Wang and Fitts (2017, 2018) also demonstrated that upregulation of ventricular K_ATP_ channel accelerates cardiac repolarization and shortens APD during stress and exercise in rats. These findings and our data suggest that an exercise protocol that prevents excessive weight gain and therefore pathological accumulation of saturated FFAs will be expected to prevent adverse potassium and calcium channel remodeling in our HFD-induced obese guinea pigs. We are currently investigating modulation by exercise of cardiac potassium channels with implications for supraventricular arrhythmias that are acquired in metabolic disorders.

Collectively, our findings revealed new directions into the dynamic functional interplay between the biophysical properties of potassium and calcium channels in supraventricular tissues, which is likely to inform development of targeted interventions toward the management of atrial arrhythmias including AF. Our data suggest that we may be able to better prevent the initiation of supraventricular arrhythmias in metabolic disorders with more effective ion channel drugs, especially with those that are targeted to *I_K_* and *I_Ca,L_*.

## Data Availability Statement

All datasets generated for this study are included in the manuscript/supplementary files.

## Ethics Statement

The animal study was reviewed and approved by IACUC, VA Healthcare System, Brooklyn, New York and Columbia University IACUC.

## Author Contributions

LM-M designed experiments and finalized manuscript. JS conceived and designed experiments, obtained funding, and finalized manuscript. AA conceived and designed experiments, analyzed results, obtained funding, and wrote the paper.

### Conflict of Interest

The authors declare that the research was conducted in the absence of any commercial or financial relationships that could be construed as a potential conflict of interest.

## Abbreviations

AF: Atrial fibrillation
APD: Action potential duration
FA: Fatty acid
hERG: Human éther-a-go-go-related gene
HFD: High-fat diet
*I_Ca,L_*: L-type calcium current
*I_K_*: Delayed rectifier potassium current
*I_K1_*: Inwardly rectifying potassium current
*I_Kr_*: Rapidly activating delayed rectifier potassium current
*I_Ks_*: Slowly activating delayed rectifier potassium current
*I_Kur_*: Ultra-rapid delayed rectifier potassium current
*I_Na_*: Large inward sodium current
*I_to_*: Fast transient outward potassium current
LFD: Low-fat diet
OA: Oleic acid
PA: Palmitic acid.
